# ZnO-NPs embedded biodegradable thiolated bandage for postoperative surgical site infection: *In vitro* and *in vivo* evaluation

**DOI:** 10.1371/journal.pone.0217079

**Published:** 2019-06-06

**Authors:** Rabia Arshad, Muhammad Farhan Sohail, Hafiz Shoaib Sarwar, Hamid Saeed, Imran Ali, Sohail Akhtar, Syed Zajif Hussain, Iqra Afzal, Sarwat Jahan, Anees-ur-Rehman, Gul Shahnaz

**Affiliations:** 1 Department of Pharmacy, Faculty of Biological Sciences, Quaid-i-Azam University, Islamabad, Pakistan; 2 Riphah Institute of Pharmaceutical Sciences (RIPS), Riphah International University, Lahore Campus, Lahore, Pakistan; 3 University College of Pharmacy, University of the Punjab, Lahore, Pakistan; 4 Department of Entomology, University College of Agriculture & Environmental Sciences, The Islamia University, Bahawalpur, Pakistan; 5 Victoria Hospital, Bahawalpur, Pakistan; 6 Department of Chemistry and Chemical Engineering, SBA School of Science and Engineering (SBA-SSE), Lahore University of Management Sciences (LUMS), Lahore, Pakistan; 7 Department of Animal Sciences, Quaid-i-Azam University, Islamabad, Pakistan; 8 School of Pharmaceutical Sciences, Universiti Sains Malaysia (USM), Pulau Pinang, Malaysia; VIT University, INDIA

## Abstract

Post-operative surgical site infections (SSI) present a serious threat and may lead to complications. Currently available dressings for SSI lack mucoadhesion, safety, efficacy and most importantly patient compliance. We aimed to address these concerns by developing a bioactive thiolated chitosan-alginate bandage embedded with zinc oxide nanoparticles (ZnO-NPs) for localized topical treatment of SSI. The FTIR, XRD, DSC and TGA of bandage confirmed the compatibility of ingredients and modifications made. The porosity, swelling index and lysozyme degradation showed good properties for wound healing and biodegradation. Moreover, *in-vitro* antibacterial activity showed higher bactericidal effect as compared to ZnO-NPs free bandage. *In-vivo* wound healing in murine model showed significant improved tissue generation and speedy wound healing as compared to positive and negative controls. Over all, thiolated bandage showed potential as an advanced therapeutic agent for treating surgical site infections, meeting the required features of an ideal surgical dressing.

## 1. Introduction

Skin is the largest organ of the integumentary system which performs six major functions i.e. sensation, thermoregulation, excretion, metabolism, non-verbal communication and most importantly protection of internal organs [1]. An injury to the skin caused by a cut, blow or any other impact resulting in laceration or breaking of skin is termed as a *wound*. Wound causes a disturbance in the defense mechanism of the skin by disrupting the epidermis, mucous membrane or underlying deep tissues of the skin [2]. According to WHO and worldwide wound incidence 2007 reporting, post-operative wounds account for the highest ratio of mortalities and infections as compared to other types of wounds. Post-operative surgical site infectious wounds may arise at the site of surgery after a month of an operation or within a year in the case of implants and infections related to the implant [3]. Surgical site infections (SSI) account for serious health-related complications along with economic burden, extended hospitalization, morbidity and mortality [4]. Surgical wound healing is a series of complex biological process that is activated by vascular constriction initiated by wounded and traumatized vessel results from myogenic spasmodic contractions of smooth muscles, localized autacoids factors from wounded tissues and involvement of nerve reflexes [5]. After constriction of smooth muscles, the wound is often sealed with platelet plug and after stoppage of bleeding, pro-inflammatory cells migrate to the wound site and initiates inflammation. Growth factors are helpful in proliferation, restoration of skin structure, eradication of pathogenic attack and remodeling at the site of injury [6]. Surgical site infections can be reduced with early healing but this can be hampered in the presence of risk factors like diabetes, obesity, smoking, and staphylococcus bacteria obstructing the epithelial growth, collagen production and delayed healing [7]. First line therapy for SSI, against *S*. *aureus* (the main causative agent for SSI), include antibiotics among which cephalosporins are the drug of choice with chances of allergic reaction with this therapy [8]. Bypassing the antibiotic therapy due to immense side effects, the treatment of surgical site infections can be successfully achieved with the local application of an ideal surgical dressing. Many chitosan and alginate-based surgical dressings, hydrogels, membranes, scaffolds, and sponges have been reported for surgical infections and are commercially available.

Among all commercially available dressings, the porous dressings are more popular owing to their moist nature and efficient wound healing capacity [9]. An ideal wound dressing for SSI must be compatible with tissues, easily applicable on the wound, moisturized enough to induce healing, mucoadhesive to bind to the application site and anti-microbial to hamper bacterial proliferation and colonization. It may also accelerate hemostasis and tissue regeneration. [10].The advent of nanotechnology has opened new avenues for designing innovative and efficient therapies to overcome the barriers faced by conventional therapies [11]. Thiolation of the polymers is a novel approach for improving the healing properties of polymers and alleviating associated side effects. Thiolated polymers are reported to have superior mucoadhesion, *in situ* gelling, poly glycoprotein (P-gp) enzyme inhibition, better control over drug release and various other biological applications *via* nanotechnology [12]. Different metal nanoparticles like silver, zinc oxide, titanium oxide, copper, copper oxide, and gold have shown tremendous antimicrobial ability alternative to antibiotics, especially against antimicrobial resistant strains [13]. These metal nanoparticles produce reactive oxygen species (ROS) which damage microbial DNA and interact with proteins and organic constituents of the microbe cause electrolyte imbalance resulting in the killing of microbes.

Here in, we report the fabrication of novel antimicrobial bandage with chitosan and ZnO nanoparticles. ZnO nanoparticles (ZnO-NPs) are being used and preferred as compared to other metallic nanoparticles, because of their specific targeting on *Staphylococcus aureus and* increased toxicity of ZnO-NPs against bacteria as compared to human cells [14]. This study also aimed at improving the physiochemical properties and therapeutic efficacy of bandage incorporated with ZnO-NPs by the covalent attachment of thiol groups on the polymer backbone. Based on the simple oxidation process and or thiol/disulfide exchange reactions (SH → S-S) within the matrix providing better tensile strength and swelling behavior [15]. The influence of immobilized thiol groups on the swelling, mucoadhesion, tensile strength, biocompatibility, and release profile was investigated *in vitro*. Moreover, wound healing potential of the bandage was evaluated in wound model induced in mice following the standard guidelines to clearly establish the superiority of newly developed medicated bandage over conventional bandages.

## 2. Materials and methods

### 2.1. Materials

Alginate (MW 8000, 60% mannuronic and 40% guluronic), Chitosan (MW 60, 000, DD 79%), EDAC (1, ethyl-3-3 dimethyl aminopropyl carbodiimide hydrochloride solution), thioglycolic acid (TGA), lysozyme and cysteine were purchased from Sigma-Aldrich Germany. Glycerol, hydrogen peroxide, iodine, safranin, crystal violet, and acetic acid were purchased from AnalaR chemicals Ltd, Poole, England. Hydroxylamine, sodium chloride, sodium hydroxide, and zinc oxide were purchased from Merck, Germany. All the solvents used were of analytical grade.

### 2.2. Methods

#### 2.2.1. Synthesis of thiolated chitosan

Synthesis of thiolated chitosan (TCS) was achieved using the reported method with slight modifications [16]. Briefly, 1% solution of chitosan was prepared in 1 M HCl. Thereafter, 500 mg of TGA and EDAC was added in the final concentration of 125 mM. The pH of the mixture was adjusted at 5.0 using 1 M NaOH solution. The reaction mixture was then incubated at room temperature under stirring for 3 h followed by dialysis to achieve purified TCS. The dialysis was performed 5 times in tubings for 3 days in total at 10°C in the dark to eliminate non-reacted TGA and for the isolation of polymeric conjugates. The sample was dialyzed one time against 5 mM HCl, and two times against 5 mM HCl with 1% NaCl. Finally, for the pH adjustment of the polymer solution to 4, polymeric conjugates were dialyzed against 1mM HCl. The purified thiolated chitosan (TCS) was lyophilized and stored at 4°C for further use.

#### 2.2.2. Synthesis of zinc oxide nanoparticles (ZnO-NPs)

Zinc oxide nanoparticles (ZnO-NPs) were prepared by the precipitation method, in which 0.2 M zinc sulfate heptahydrate and 0.5 M NaOH were used initially as precursors. 0.2 M zinc sulfate heptahydrate was dissolved in distilled water and stirred for 15 min. Then 0.5 M pre-heated NaOH solution was added to the solution within 3 seconds, heated at 60°C and stirred for about 1 h. The color of the mixture changed from clear white to turbid due to precipitation and followed by probe sonication for 5 min. After this, centrifugation at 13500 rpm and 25°C was done for 45 min. The pellet obtained was resuspended in a small amount of water and sonicated again for 5 min, producing ZnO-NPs [17, 18].

#### 2.2.3. Synthesis of thiolated chitosan-alginate (TCS-Alg) bandage

Thiolated chitosan-alginate (TCS-Alg) bandage was synthesized by mixing different ratios of alginate and TCS gel [19]. 3% (w/v) alginate gel was prepared and heated at 60°C under continuous stirring for 15 min. TCS (1%, w/v) was dissolved in 1% HCl followed by precipitation with 1% NaOH solution resulting in TCS gel. Then TCS gel was further mixed with alginate hydrogel to produce a TCS-Alg gel. The mixture was homogenized for 5 min at 24000 rpm to obtain a smooth TCS-Alg gel. Previously synthesized ZnO-NPs were added in the hydrogel and homogenized for 30 min to prepare ZnO-NPs loaded gel. The final gel mixture was lyophilized for 10 h to obtain flexible and porous bandages.

#### 2.2.4. Quantification of immobilized thiol groups

Thiol group immobilized to the chitosan backbone was quantified *via* spectrophotometry using Ellman’s reagent as reported previously [16, 20]. Briefly, 0.5 mg of each CS and TCS was hydrated in 250 μL of deionized water. 500 μL of freshly prepared Elman’s reagent and 250 μL of phosphate buffer (pH 8.0, 0.5 M) were added into the polymeric suspension. The mixture was incubated at room temperature for 3 h followed by centrifugation and the supernatant was transferred to a 96-well plate. The absorbance was measured at a wavelength of 430 nm in a microtiter plate reader (PerkinElmer, USA). TGA standard was used to calculate the Immobilization of thiol group contents on chitosan.

#### 2.2.5. Determination of disulfide bonds

Disulfide content was determined through a reported method [16]. About 0.5 mg of each CS and TCS was hydrated in 350 μL of deionized water and to this 650 μL of phosphate buffer (pH 6.8, 0.05 M) was added. 1% (m/v) of freshly prepared sodium borohydride was added after 30 min. After incubation of mixture for 1 h at 37°C, 200 μL of HCl (5 M) was added to decompose remaining sodium borohydride. Neutralization of the solution was done by adding 1 m phosphate buffer (pH 8.5, 1 M) along with phosphate buffer having 100 μL Ellman’s reagent (0.4%) immediately followed by incubation for another hour. From this 300 μL was transferred to microplate and absorbance was measured at 430 nm using a microtitre plate reader (PerkinElmer, USA). Subtraction of the calculated thiol group in the earlier step from the total immobilized thiol groups on TCS will give free thiol groups [21].

#### 2.2.6. Particle size, polydispersity index (PDI), and zeta-potential analysis

The particle size, zeta potential and PDI of the ZnO-NPs was measured using Nano-ZS (Malvern Instruments, Inc., MA, USA).

#### 2.2.7. Surface morphology and appearance

Scanning electron microscope (SEM) was performed to study the morphology of bandages, whereas, transmission electron microscopy (TEM) was employed for the analysis and size determination of ZnO-NPs. The analysis was performed using (FEI Nova Nano SEM 450, USA) equipped with transmission electron microscopy. Samples were carefully placed on the carbon-coated copper grid and followed by smudging with a drop of 1% ammonium molybdate solution.

#### 2.2.8. FTIR, DSC and XRD analysis

Physicochemical stability and electrostatic interactions of ZnO-NPs with TCS-Alg in bandage were studied *via* different analysis techniques. FTIR *via* (Bruker α, USA), was obtained by KBr disk method in the range of 4000–500 cm^-1^. Powder X-ray diffraction *via* (Bruker, D2 Phaser, USA) was obtained by operating the instrument between θ of 20°-70° with a step size of 0.5505 and ƛ_(Cua-k)._ Differential scanning calorimetry (DSC) and Thermogravimetric analysis (TGA) *via* (TA Instruments, SD Q600, USA) was performed on different formulations over a temperature range of 50–300°C with a heating rate of 10°C per min under air purging at 10 mL/min respectively [16].

#### 2.2.9. The analysis of bandage porosity

Alcohol displacement method was applied to determine the porosity of the bandage. In this method, bandages (both with ZnO-NPs loaded and without ZnO-NPs) having weight 10 mg with an area 1 x 1 mm, was immersed in 1 ml ethanol, just enough to saturate the bandages [22]. After 24 h the polymers were taken out and weighed. Porosity (P) was calculated by the formula:

$$P\;=\;\frac{W2-W2}{\rho V1}$$

Where W1 indicates the weight of the bandages before immersing; while W2 is the weight after immersing; V1 is the volume of alcohol before immersing; *ρ* is the density of alcohol which is a constant.

#### 2.2.10. Estimation of swelling ratio of the bandage

Bandage pieces (both with ZnO-NPs loaded and without ZnO-NPs) having weight 10 mg and area 1 x 1 mm were immersed in simulated wound fluid at 37°C. The bandage pieces were taken out after 3, 6, 18 and 24 h and weighed accordingly. Swelling ratio was calculated using the formula:

$$DS\;(degree\;of\;swelling)\;=\;\frac{Ww-Wd}{Wd}\;X\;100$$

Where Ww is the wet weight of the bandage pieces and Wd is the dry weight of the bandage pieces [23]

#### 2.2.11. Mucoadhesion studies of the bandage

Mucoadhesion studies of the bandage were performed *via* disintegration apparatus following the method reported [24]. Basket rack assembly was filled with simulated wound fluid and uniform surface of basket rack assembly was achieved by covering it with aluminum foil. Freshly excised goat skin was attached to basket rack assembly with the help of thread. The bandage pieces were hydrated on one side and were brought in contact with the goat skin individually and the pressure was applied for the proper attachment [25] [26]. The basket rack assembly was then vertically fixed and allowed to move up and down in such a way that the bandage was completely immersed in the simulated wound fluid at the lowest point and was out of it at the highest point. The time taken by complete separation of skin because of bandage degradation was recorded.

#### 2.2.12. Evaluation of tensile strength of the bandage

The tensile strength of the dried bandage was determined using tensile strength apparatus (Presto, India) at room temperature at a constant displacement rate of 1 mm/min. The dried bandage was clamped at both ends of tensioner with fixtures and fixtures were pulled slowly on the bandage sample until it broke. Tensile strength was calculated using the formula given below through the applied load at rupture divided by the cross-sectional area of the bandage:

$$\text{Tensile}\;\text{strength}=\frac{Load\;at\;failure\;X\;100}{Strip\;thickness\;X\;strip\;width}$$

#### 2.2.13. *In vitro* lysozyme biodegradation of the bandage

The *in-vitro* lysozyme degradation of bandage was based on the reported method [19]. Briefly, bandages were weighed (10 mg, 1×1 mm) and immersed in lysozyme (10,000 U/mL) containing medium and incubated at 37°C for 21 days. Original weight of the bandage was noted as Wi. After 7, 14 and 21 days, each set of bandages were taken out from the PBS containing lysozyme, washed with deionized water to get rid of the ions adsorbed on the surface followed by freeze-drying. The dry weight was noted as Wi and wet weight was noted as Wt. The rate of degradation of bandage was calculated using the formula:

$$Degradation\;\left(\%\right)=\left[\frac{\text{Wi}-\text{Wt}}{\text{Wi}}\right]\;x\;100$$

#### 2.2.14. ZnO-NPs release studies from the bandage

The release of ZnO-NPs from both bandages was conducted in paddle over disk dissolution apparatus (Erweka, Germany). Dissolution vessels of paddle over disk were filled with 500 mL of simulated wound fluid (pH 7.4) equilibrated at 37 ± 0.5°C. Weighed quantities of bandages equivalent to 5mg were attached to the stainless-steel disk assembly by double-sided adhesive tape. Stainless steel disk assembly holding the bandage pieces was centered using a glass rod and placed at the bottom of the vessel with release surface facing up [27]. The stirring speed was maintained at 150 rpm and samples of 5 mL were collected at specified time intervals for 72 h. The withdrawn sample was replaced with the same amount of fresh media equilibrated at 37± 0.5°C The samples were analyzed by atomic absorption spectroscopy. DD solver, (a free Microsoft add-in programme) was used to analyze the release mechanism of ZnO-NPs from bandages.

#### 2.2.15. Biological characterization

All the animal investigations were performed as per the requisite protocol approved by the Bio-Ethical Committee of Quaid-i-Azam University Islamabad, Pakistan (Protocol No. DFBS/2016-551/BECFBS-QAU-83) considering the EU directives 2010/63 for animal studies and Arrive guidelines. The swiss albino mice of either sex, weighing 30 ± 5 g were obtained from the Department of Animal Sciences, QAU, Islamabad. The animals were housed is separate metal cages under adequate conditions maintaining temperature at 22 ± 2°C, humidity 40–45% and 12 h light and dark cycle. All animals were given their specified food palettes with free access to water.

#### 2.2.16. Coagulation testing of the bandage

The coagulation potential of the bandage was evaluated *via* blood coagulation analysis. Fresh human blood (with volunteer consent) was collected in a vial containing an anticoagulant. The blood was then poured onto bandages followed by addition of calcium chloride. The bandage samples were incubated in a shaking incubator at 37°C for 30 min. Optical density was then checked at 540 nm to evaluate the release of free hemoglobin from red blood cell (RBC) membrane lyses [19].

#### 2.2.17. *In vitro* antibacterial activity for identification of bacteria

*In vitro*, the antibacterial efficacy of the bandage was determined by zone inhibition method. The mice were anesthetized by using chloroform, and the wound was created by sterilized needles. The sterilized swab was immediately placed on wound thus carrying wound exudates along with it and these carried wound exudates were cultured in LB broth. Following dissolution, the broth was incubated at 37°C for 24–48 h. Bacteria were then streaked on LB agar plates separately for determination of shape and biochemical assays including gram staining, catalase, coagulase and oxidase [28]. The cultured plates were treated with both bandages, and zones of inhibition were measured to see the antibacterial effect.

#### 2.2.18. Cell viability evaluation

Cell viability was assessed on Hela cell lines (ATCC, CCL2, Homo sapiens) using the MTT assay. Samples of the ZnO-NPs and bandages were sterilized by ethylene oxide gas and subjected to 12-well cell culture plates. Hela cells were trypsinized and seeded on the samples of ZnO-NPs and bandages. After seeding, the substrate mixture of Hela cell lines bandages was incubated at 37°C for 72 h and then Almar blue was added at specified time intervals. After the incubation period, the cell viability was measured by determining the optical density at 570 nm using a microplate spectrophotometer (PerkinElmer, USA).

#### 2.2.19. *In vivo* wound healing capacity of the bandage

*In vivo* wound healing capacity of the bandage was assessed on the murine model. The mice were randomly divided into 4 groups (n = 6). Group 1 served as control which remained untreated, whereas group 2, 3, and 4 were treated with a marketed bandage, CS-Alg-ZnO, and TCS-Alg-ZnO bandages respectively. The mice were carefully monitored daily for any abnormal changes in mice and chances of infection and after every seven days for the wound healing process. All the mice were anesthetized through isoflurane (3–5% with oxygen) [29], afterward, the wound was created by sterilized needles near the hind limb. The bandages were applied to the wound and remained there All the mice were monitored daily for the size of the wound and healing process. This monitoring process was continued for 28 days. The untreated group was given analgesics and antibiotics to cure the infective wound after the completion of study [6, 30].

#### 2.2.20. Histological evaluation of skin

Histopathological changes, in mice, wounded and healed skin tissue were microscopically examined from stained slides of skin tissues. The skin tissues slides were fixed with 10% formalin solution and further processed with alcohol and xylene. After processing, these slides were embedded in paraffin wax and prepared with a microtome. In the end, the slides were stained with hematoxylin and eosin to observe microscopically the histological changes in the skin [31].

#### 2.2.21. Biodegradation of the bandage

Biodegradation of the bandage was accessed on the murine model. The mice (n = 5), weighing 20–25 g, were selected and anesthetized *via* chloroform. The hair was trimmed to expose the subcutaneous layer and the wound was carefully introduced on the skin. The bandage was carefully applied on all mice skin to see the biodegradation of bandage. Biodegradation was monitored at day 7, 14, 21 and 28 by visual observations and through SEM analysis by removing the bandage from mice on a respective day for observing the structural changes.

#### 2.2.22. Statistical analysis

Statistical analysis of all the parameters was performed using t-test on raw data (Excel, Microsoft Office 2016) keeping significance level *p* < 0.05.

## 3. Results and discussion

### 3.1. Synthesis and characterization of TCS

Thiolated chitosan was successfully prepared *via* EDAC coupling method based on carbodiimide chemistry. The lyophilized thiolated chitosan appeared as a white fibrous powder with improved aqueous solubility. The number of thiol groups immobilized on chitosan was calculated to be 161 ± 7 μM/g and the number of disulfide bonds was calculated to be 96 ± 9 μM/g of TCS which exhibited successful thiolation of the chitosan.

### 3.2. Synthesis and characterization of ZnO-NPs

ZnO-NPs were successfully synthesized *via* precipitation method. The particles size was found to be 61 ± 9.29 nm. The polydispersity index (PDI) is an indicator of uniformity of synthesis and value below 0.5 indicates a uniform synthesis of nanoparticles. ZnO-NPs showed PDI 0.310 ± 0.01 indicating uniformity in synthesis. Zeta potential is an important indicator of the stability of nanoparticulate dispersed systems. For nanoparticles, high zeta potential value is preferred whether positive or negative. The zeta potential of ZnO-NPs was + 20.27 ± 2.92 meV.

### 3.3. Synthesis and characterization of the bandages

**Fig 1:**
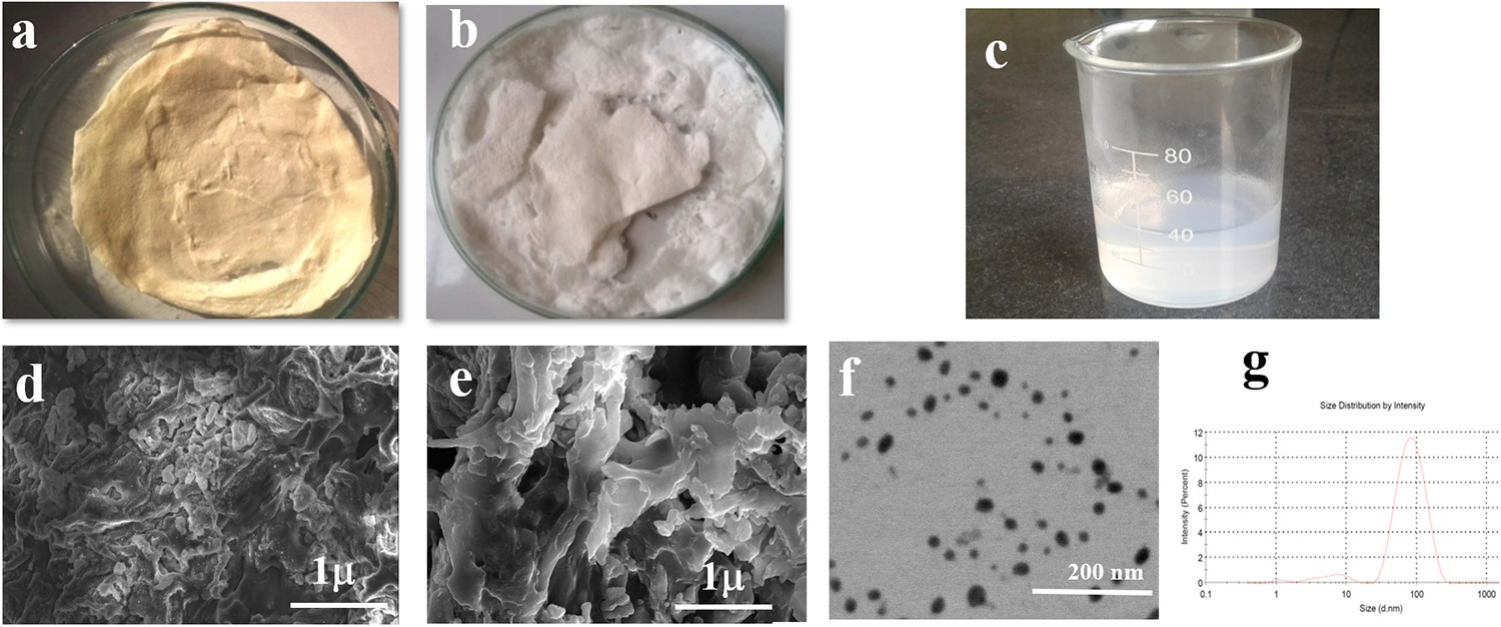
Physical appearance and electron microscope analysis of bandages. **(A)** lyophilized CS-Alg-ZnO bandage, **(B)** lyophilized TCS-Alg-ZnO bandage, **(C)** lyophilized ZnO nanoparticles. Whereas, **(D)** SEM analysis of CS-Alg-ZnO bandage, **(E)** SEM analysis of TCS-Alg-ZnO bandage, **(F)** TEM analysis of ZnO nanoparticles and **(G)** size distribution histogram of ZnO nanoparticles.

Two different types of bandages based upon CS and TCS along with alginate and ZnO-NPs were prepared as CS-Alg-ZnO and TCS-Alg-ZnO respectively. The bandages appeared as a white fibrous sheet-like structure with sufficient porosity as shown in Fig 1.

### 3.4. Electron microscopic analysis of ZnO-NPs and bandages

SEM analysis was performed for the morphology evaluation of ZnO-NPs and different bandages. The SEM images of the bandage (Fig 1A and 1B) showed fibrous appearance due to the TCS/Alg, resulted in the development of the disulfide linkages between the thiol groups of polymeric chains and cotton fibers present in the bandage that impart fibrous nature to the lyophilized thiolated polymers/bandage. The porous nature thus they will be suitable for wound healing. ZnO-NPs appeared with varying morphology with a smooth surface (Fig 1C). The ZnO-NPs were observed through transmission electron microscope (TEM), showing mostly the clusters of ZnO-NPs insides the bandage due to the increased viscosity of bandage owing to viscous polymers matrix of TCS and alginate [10]. As DLS measurements are done by diluting the sample in de-ionized water after sonication and further such measurements are not known to have any clue of presenting the morphology of particles except hydrodynamic size. It is thus, the DLS measurements give an idea about the size of solid metal nanoparticles along with the soft boundaries of ligands at their peripheries. The TEM analysis was performed to check the actual size of the ZnO-NPs which showed particle size of 37 ± 4 nm.

### 3.5. FTIR, XRD, DSC and TGA analysis

**Fig 2:**
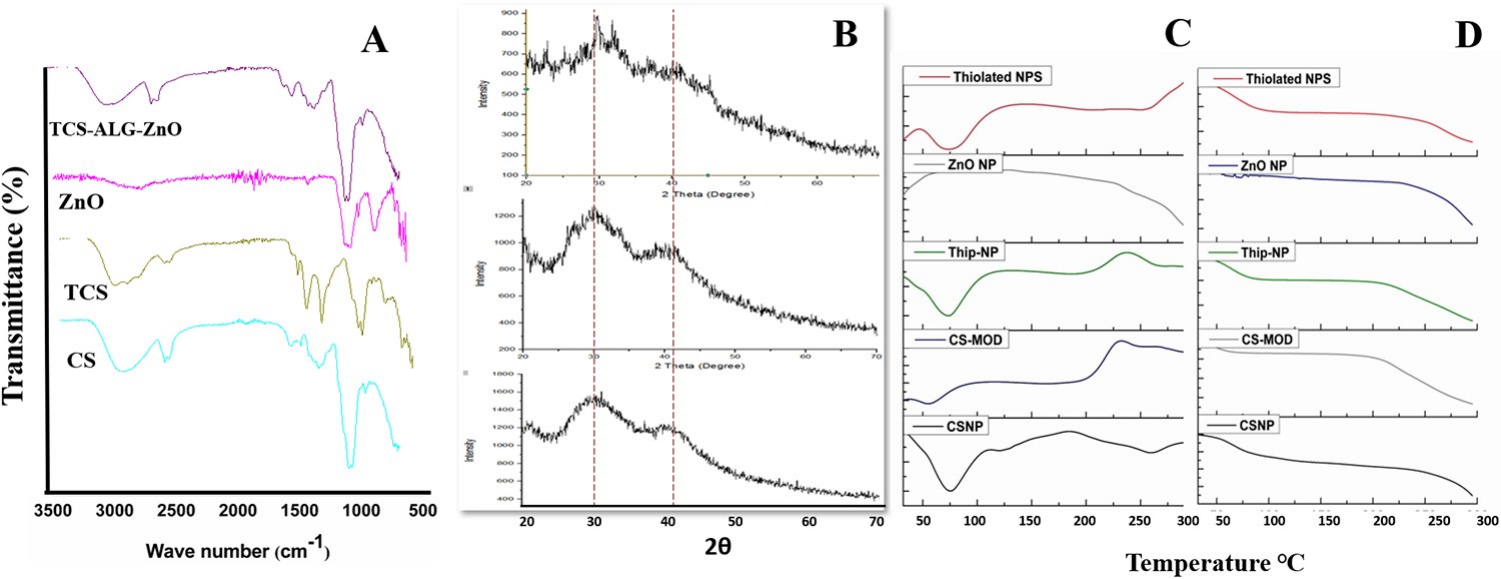
Physicochemical compatibility analysis conducted using various techniques. **(A)** FTIR of polymers, ZnO-NPs and TCS-Alg-ZnO; **(B)** XRD patterns of non-thiolated, thiolated bandage as well as ZnO NPs; **(C)** DSC of polymers, bandages and ZnO-NPs; and **(D)** TGA of bandages and ZnO -NPs.

The stability of the drug and its functional groups inside different polymers were studied through Fourier Transformed Infrared (FTIR) spectroscopy and the results are shown in Fig 2A. The spectrum of CS clearly showed absorbance bands at 1654 cm^-1^ (amide I), 1604 cm^-1^ (NH_2_) bending and 1382 cm^-1^ (amide III). The band at 1156cm^-1^ (asymmetric stretching of COOOC bridge), 1072 cm^-1^ and 1023 cm^-1^ (skeletal vibration because of -COO stretching) are important features of its saccharin structure. However, in the TCS spectrum peaks at 3351 cm^-1^ and 3209 cm^-1^ represent O-H and N-H stretching. And a new peak is observed at 1630 cm^-1^ is assigned to the acylamino group. Also, the peak around 1607 cm^-1^ decreased, indicating that amino groups are partly conjugated to TGA [32]. FTIR peaks at 3290 cm^-1^ also represent the stretching vibrations of ‒OH and ‒COOH group of sodium alginate. The appearance of small peaks after 1000 cm^-1^ which appeared in a thiolated bandage but are absent in unmodified bandage represents the incorporation ZnO-NPs in the thiolated bandage.

The XRD pattern in Fig 2B confirmed the crystalline nature of ZnO-NPs. But bandage embedded with ZnO-NPs showed amorphous nature because of the amorphous nature of polymers present in the bandage. In TGA analysis, ZnO-NPs showed an exothermic peak (crystalline) at 55°C while endothermic peak (amorphous) was observed at 250°C, similar was the result with unmodified chitosan. TGA results (Fig 2C) of both bandages showed the disordered crystalline state at 300°C resulting in weight loss because of the vaporization of moisture and degradation of polymers at this temperature DSC (Fig 2D) determined the crystalline and amorphous or disordered crystalline state of polymeric bandages along with their physicochemical properties after modifications. [30].

### 3.6. Analysis of the bandage porosity

**Fig 3:**
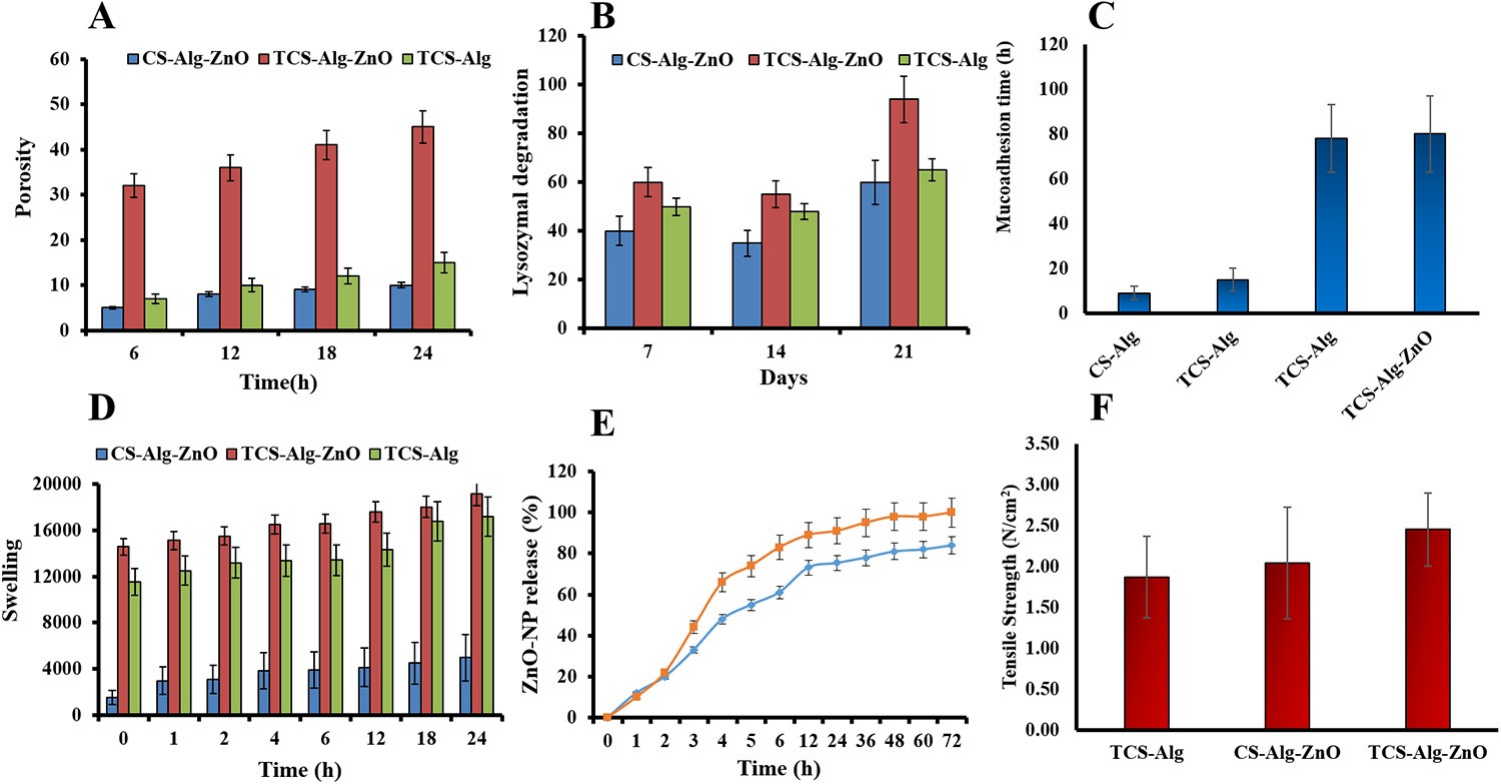
Characterization of various bandages through physicochemical parameters. **(A)**The porosity analysis of the TCS-Alg-ZnO, CS-Alg-ZnO and TCS-Alg bandages; **(B)** Lysozyme degradation profile of TCS-Alg-ZnO, CS-Alg-ZnO and TCS-Alg for 21 Days; **(C)** mucoadhesion study of TCS-Alg-ZnO, CS-Alg-ZnO and TCS-Alg bandages; **(D)**, Swelling behavior of TCS-Alg-ZnO, CS-Alg-ZnO, CS-Alg and TCS-Alg bandages; **(E)**, *In vitro* release of ZnO-NPs from TCS-Alg-ZnO and CS-Alg-ZnO bandages; and **(F)** Tensile strength of TCS-Alg-ZnO, CS-Alg-ZnO and TCS-Alg bandage. All the results are presented as mean ± SD of triplicate experiments and the significance level was considered for *p* < 0.05 for all statistical analysis.

Porous bandages are the primary requirement for ideal wound healing because of their capability of rehydrating themselves quickly in the presence of mucoadhesive polymers [33]. Pores are produced in a bandage during slow and gradual lyophilization of the polymeric matrix. The porosity of the TCS-Alg-ZnO bandage depends upon the pore size and facilitates the absorption of the exudates from the surgical wound site and results in the decrease in the rate of colonization of skin by *Staphylococcus aureus* [34]. Therefore, it supports healing, cellular organization, and angiogenesis of the tissues. Porosity increases the mobility and stability of the ZnO-NPs and enhances their diffusing capacities through the polymeric matrix in the release medium, thus helpful in maintaining effective and control drug release. Increased porosity also contributes to enhanced swelling and mucoadhesion [19]. The results of porosity analysis are shown in Fig 3A. The porosity of CS-Alg-ZnO bandage was 5%, while for TCS-Alg-ZnO bandage it increased to 45% that might be owing to the presence of disulfide linkage of TCS and presence of ZnO-NPs in the polymeric mesh of the bandage.

### 3.7. Lysozyme degradation of the bandage

Lysozyme degradation profile for 21 days (Fig 3B) showed that TCS-Alg-ZnO mucoadhesive bandage possesses the innate ability of lysozymal degradation so that the bandage can be self-degraded thus no need to change or remove it. Moreover, the thiolated bandage also possesses increased polymeric strength and ability to withstand mechanical pressure. Due to these properties; it might be helpful in increasing the rate of dry mass loss of bandage thus resulting in faster and efficacious biodegradation as compared to the CS-Alg-ZnO. Moreover, other features like higher swelling and porosity also account for increased biodegradation by exposing the N-acetyl glucosamine of TCS for the lysozyme attack. All the degradation products are non-toxic in nature which further supports its wide application [35].

### 3.8. Mucoadhesion studies of the bandage

The limitation with conventional bandages is that the continuous exudate secretions from wound make it difficult for the bandage to remain intact and cover wound properly over a long period. Moreover, as wound healing progresses, it is difficult for a bandage to remain intact and cover the area for proper wound healing by providing necessary moisture. Also, these bandages do not have any antimicrobial agent which require the application of antimicrobial agent prior to application. Mucoadhesive drug delivery systems have emerged as a very appealing approach to extend the residence time of the formulation at the delivery site due to which the bioavailability is usually increased. Thiomers are introduced as a new and promising family of mucoadhesive polymers for drug delivery [15]. The key feature of the developed TCS-Alg-ZnO bandage might be the associative interaction between bandage and skin epithelial mucin and results in interchain interlocking, conformational changes and chemical interaction [36]. Mucoadhesion involves the attachment of mucoadhesive polymer at skin mucus which is glycoproteins which develops disulfide linkage with TCS-Alg-ZnO bandage [37]. Mucoadhesion is not only confined to the adherence at the wound site and causing skin eruptions but also hydration is complementary for a mucoadhesive polymer to expand and to induce mobility in the polymer chains in order to enhance the interpenetration process between polymer and mucin. Further, the anionic glycosaminoglycan (GAG) in the extracellular matrix of the wound can electrostatically attract cationic TCS and ZnO-NPs, leading to better mucoadhesion and accumulation of ZnO-NPs [38]. Enhanced TCS-Alg-ZnO swelling permits a mechanical entanglement by exposing the bioadhesive sites for hydrogen bonding and/or electrostatic interaction between the TCS-Alg-ZnO and the mucus network [39]. Prolonged mucoadhesion of 72 h with TCS-Alg-ZnO as compared to 6 h with CS-TAlg-ZnO (Fig 3C) suggested that the TCS-Alg-ZnO bandage will remains attached to the wound bed irrespective of movement of the object for a longer period of time and will not hinder the skin movement and detachment. As all polysaccharide based polymeric components of the bandages are biocompatible, highly absorptive and non-occlusive [40]. Moreover, TCS-Alg-ZnO rehydration and bactericidal environment due to increased porosity, swelling and antimicrobial potential is responsible for hydrated mucoadhesion (thus preventing the dried sticking of the bandage at the wound site) and efficient wound healing [41].

### 3.9. Swelling studies of the bandage

The swelling features of the bandage are responsible for enhanced mucoadhesion, drug release and stability. Strengthened adhesion could be achieved when the bandage gets attached to the mucus membrane [24]. Following skin attachment, the bandage is swelled by absorbing wound exudates through diffusion and capillary action from the underlying mucosal tissue and results in the efficient transfer of oxygen and nutrients for wound healing. Adequate swelling ensures the nourishment of dried wound by transfer of oxygen and nutrients for wound healing. Moreover, as reported in the present study that the dry wound of the mice was nourished with adequate swelling due to thiolation and increased mucoadhesiveness [2]. Swelling is also responsible for seeding of cells and distribution of cells through polymers of the bandage. Swelling of the bandage was observed in simulated wound fluid pH 7.4 [42]. The results of bandage swelling (Fig 3D) showed the highest swelling rates of TCS-Alg-ZnO bandage owing to the presence of surface thiol groups and disulfide linkages which possess a higher ability to absorb wound exudates.

### 3.10. ZnO-NPs release studies

ZnO-NPs entrapped in the bandage must be released to produce the anti-microbial effect locally. TCS is reported for controlled/sustained release of various loaded drugs [43]. Release studies of ZnO-NPs from thiolated bandage were studied for 72 h and results depicted that around 70% of ZnO-NPs as shown in Fig 3E. The data showed sustained release properties from TCS-Alg-ZnO as compared to CS-Alg-ZnO. TCS-Alg-ZnO bandage at first released ZnO-NPs slowly due to intramolecular strong disulfide bonds, while along with passage of time and good swelling and porosity properties voids were created and ZnO-NPs release was improved. Different kinetic models were applied to the release data to check the mechanism of ZnO-NPs release. The release pattern from TCS-Alg-ZnO was best fitted into *Korsmeyer-Peppas* kinetics (Table 1) with a maximum *R*^*2*^ value of 0.86 with *n* value 0.27. From n values, it can be concluded that it followed (Fickian) diffusion mechanism i.e. gradual swelling of the TCS bandage released the ZnO-NPs *via* diffusin through polymer matrix [44]. From CS-Alg-ZnO release followed first order release mechanism with a maximum ***R***^***2***^ value of 0.93 and ***K***_***1***_ value of 0.01 [45].

**Table 1:** Dissolution data models were applied to determine the ZnO-NPs release kinetics from bandages, where A = TCS-Alg-ZnO and B = CS-Alg-ZnO.

Zero Order			First Order		Korsmeyer-Peppas		Higuchi		Hixon-Crowell	
R^2^		K_0_	R^2^	K_1_	R^2^	n	R^2^	K_H_	R^2^	K_HC_
**A**	0.74	1.22	0.10	0.02	0.86	0.27	0.76	9.79	0.03	0.07
**B**	0.82	1.55	0.93	0.01	0.98	1.00	0.82	12.6	0.86	0.01

### 3.11. Evaluation of tensile strength of the bandage

The results of tensile strength (Fig 3E) of bandage depicted that TCS-Alg bandage is stronger as compared to CS-Alg bandage because of increased flexibility, folding endurance and strength of TCS as compared to that of CS. TCS-Alg bandage showed a tensile strength of about 3.5 N/m^2^ which is very ideal for the successful application and stress-bearing capacity of the TCS-Alg-ZnO bandage. The CS-Alg bandage showed less tensile strength values i.e. 1.5 N/m^2^. Addition of ZnO-NPs in TCS-Alg-ZnO bandage resulted in bit decrease in tensile stress because of their crystalline nature i.e. 2.5 N/m^2^ which is again good for achieving stability, efficacy, and strength [22].

### 3.12. Coagulation testing of the bandage

**Fig 4:**
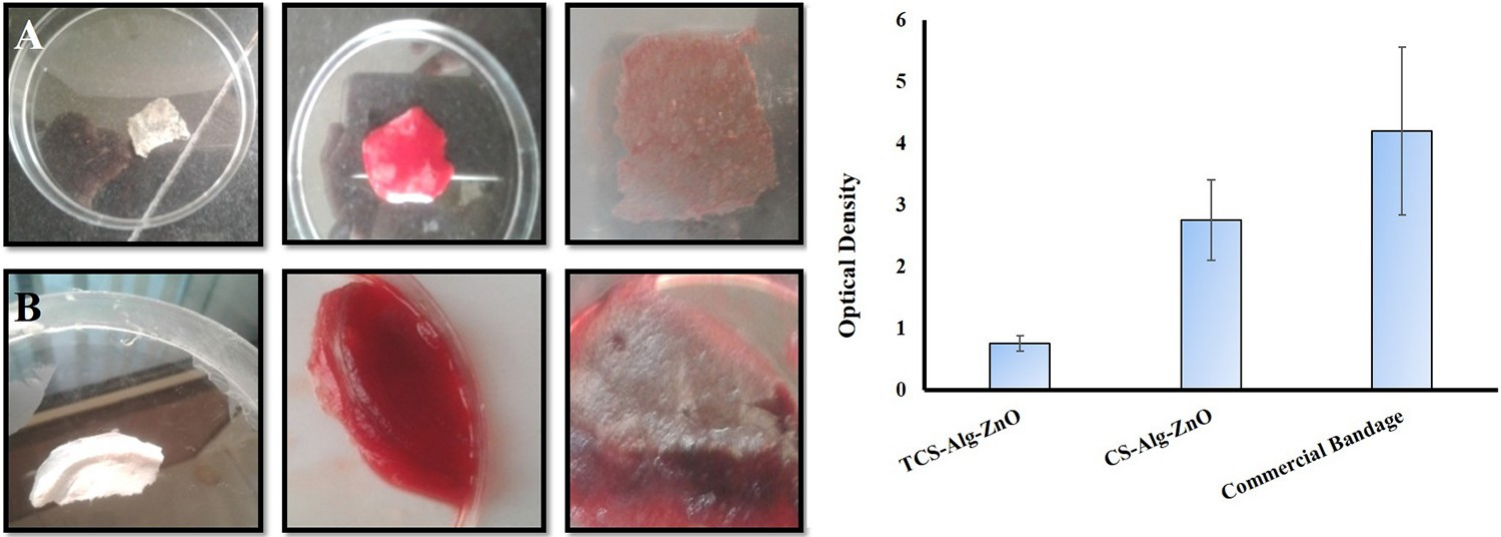
Coagulation and hemostasis testing of bandages with fresh human blood. **(A)** panel showing coagulation test for TCS-Alg-ZnO bandage, **(B)** panel showing coagulation test against CS-Alg-ZnO bandage. **(C)** comparison of improvement in hemostasis in the presence of TCS-Alg-ZnO as compared to CS-Alg-ZnO and TCS-Alg. The results are shown as Mean ± S.D (n = 3) and TCS-Alg-ZnO bandage with *p < 0.01 as compared to both bandages.

The results of coagulation testing are shown in Fig 4. At 540 nm, the lower optical density value of TCS-Alg-ZnO bandage as compared to CS-Alg-ZnO and marketed bandage showed the lower release of free hemoglobin from red blood cell (RBC) membrane lysis. Hence results proved the superior blood clotting ability of TCS-Alg-ZnO bandage as compared others. Increased coagulation resulted due to the strong interaction between the positively charged amine group of TCS negatively charged blood cells [18]. Increased coagulation is ideal for healing and blood stoppage of surgical bleeding infections.

### 3.13. *In vitro* antibacterial activity of the bandages

**Fig 5:**
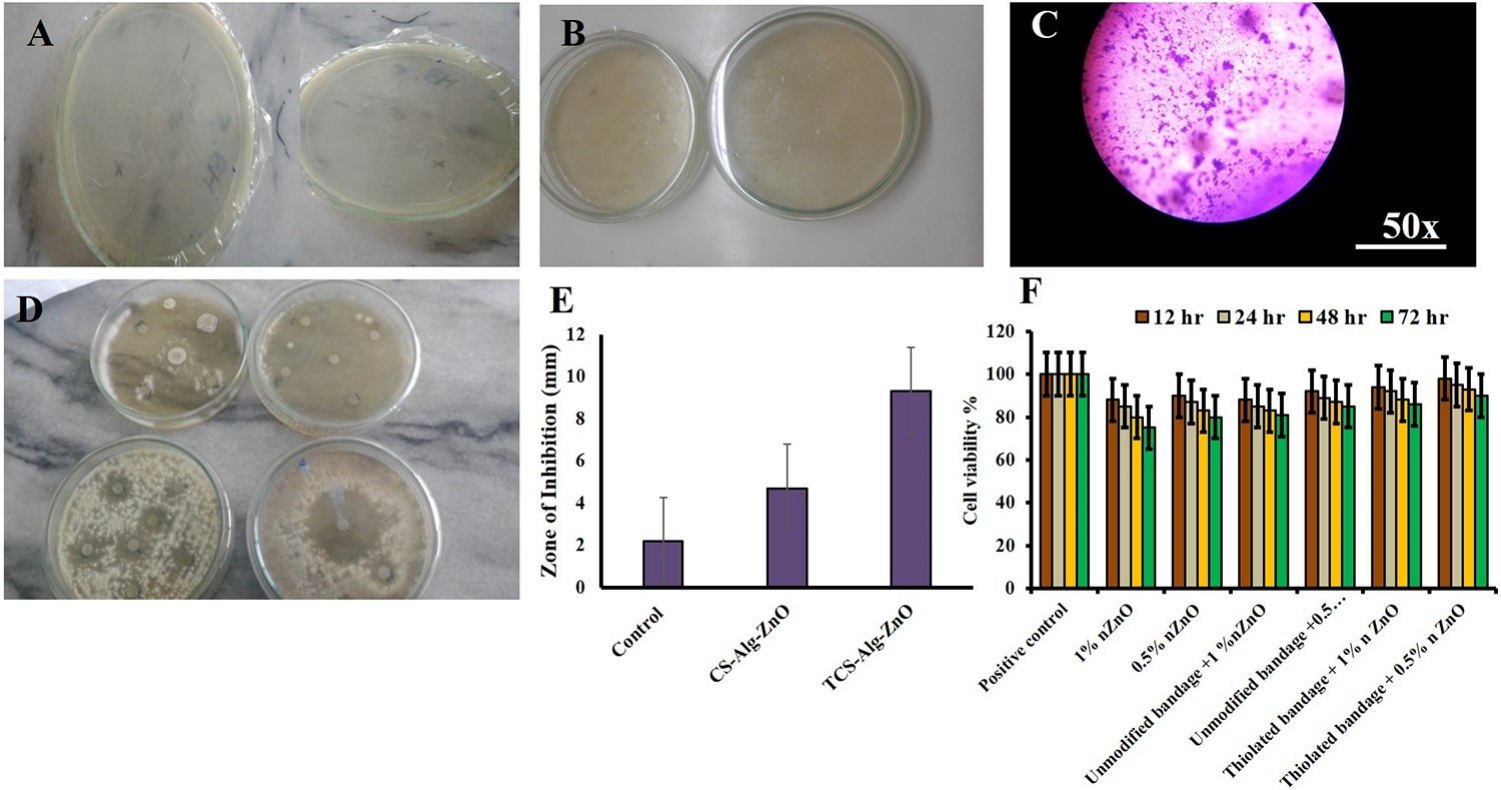
Antibacterial activities of bandages. **(A)** inoculated agar plates**; (B)** agar plates having TCS-Alg-ZnO bandages; **(C)** Microscopic view of stained bacterial culture from incubated Petri dishes having TCS-Alg-ZnO bandages; **(D)** Zone of inhibitions by different bandages and control group; **(E)** Comparison of the zone of inhibitions by different bandages and control group **(F),** Cell viability (%) against different concentrations of the bandages and ZnO-NPs.

The ZnO-NPs exhibit a complex mechanism of antimicrobial activity produced by the production of reactive oxygen species (ROS), H_2_O_2_ and Zn^+2^ which interact with microbial cell wall and intracellular biomolecules resulting in cell death [46]. However, in the case of NPs-cell wall interaction, particle size plays a vital role as smaller the particles size of the ZnO NPs, greater will be the surface area providing increased contact with the microbes resulting in enhanced antimicrobial effect [47]. The *in-vitro* antibacterial activity of the bandage was determined by applying the different biochemical test to the wounded bacterial culture i.e. gram-staining, disk diffusion method *Coagulase*, *Catalase*, *DNAse*. Results in Fig 5 showed the presence of *staphylococcus* bacteria majorly in wound cultures. Disk diffusion method resulted in an increased zone of inhibition of TCS-Alg-ZnO bandage as compared to CS-Alg-ZnO bandage and control group. Thus, it is evident that TCS-Alg-ZnO bandage will be preferable in all type of skin infections due to highly targeted drug delivery properties in combination with enhanced antimicrobial action of 38 nm sized ZnO-NPs [48].

### 3.14. Cell viability studies

Cell viability of the ZnO-NPs and both bandages was conducted using Hela cell lines. It was evident from results that the cell viability of ZnO-NPs and bandages was time and dose-dependent. The cell viability of the 1% ZnO-NPs was 88% at 12 h and decreased up to 75% in 72 h. 0.5% ZnO-NPs showed 90% cell viability at 12 h and 80% viability up to 72 h. Similarly, above mentioned concentrations of TCS-Alg-ZnO resulted in increased cell viability by up to 90% as compared to ZnO-NPs. CS-Alg-ZnO bandage resulted in cell viability up to 85%. Results concluded that ZnO-NPs decreased the cell viability might be due to the generation of reactive oxygen species (ROS) but this issue can be resolved by incorporating the ZnO-NPs in the thiolated bandage resulting in maximum cell viability. Thiolated bandage embedded with ZnO-NPs increased cell viability because it possesses innate abilities to minimize toxicity and enhancing the stability of the formulation.

### 3.15. *In-vivo* wound healing capacity of the bandage

**Fig 6:**
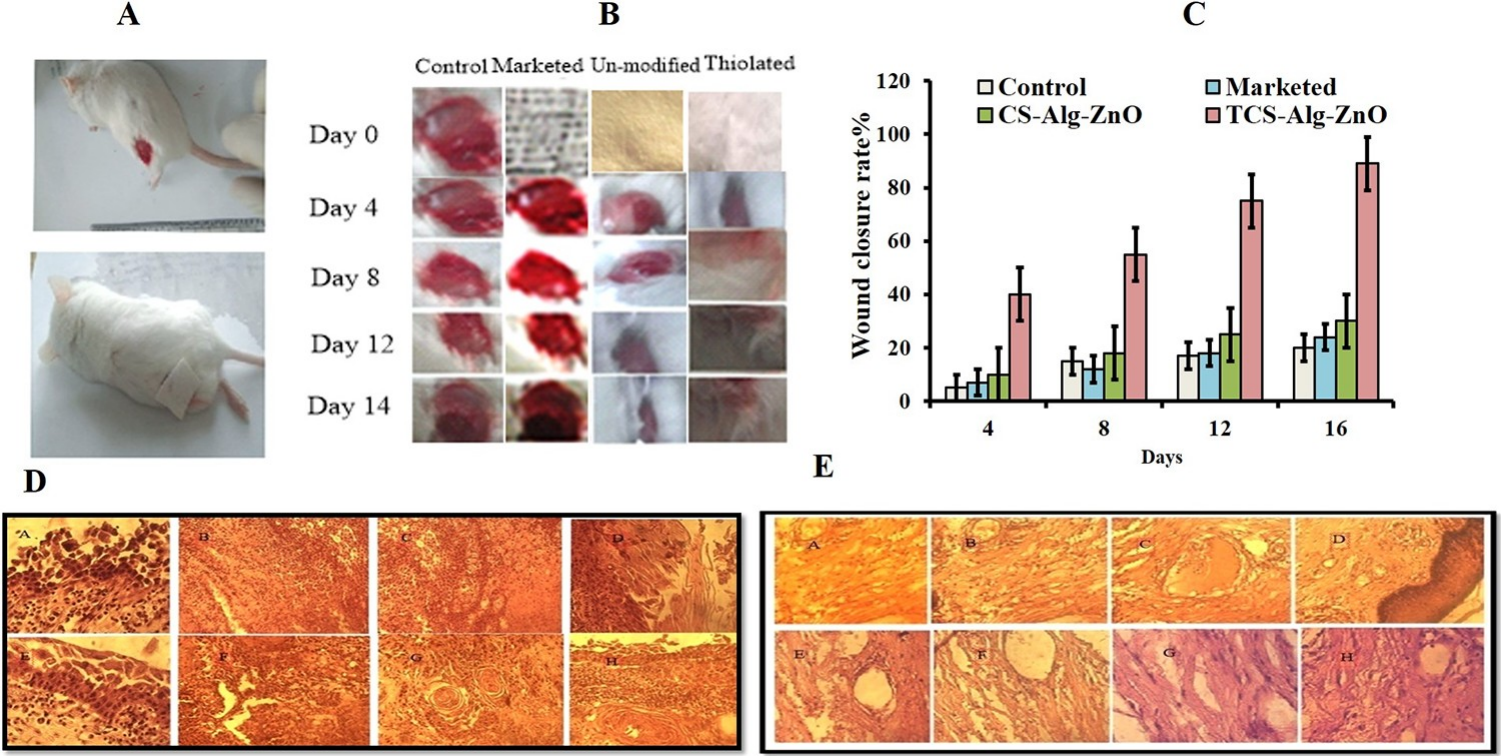
*In-vivo* analysis of wound healing ability of bandages. **(A)** mice with induced wound and applied bandage on wound; **(B)** panel showing 28 days study on wound healing process in the presence of TCS-Alg-ZnO, CS-Alg-ZnO and commercial bandages; **(C)** graph showing speed of wound closure in terms of reduction in wound size after application of bandages; **(D)** Histopathological panel showing skin tissue from a wound site at various time intervals and **(E).** Histopathological evaluation showing skin regeneration after application of TCS-Alg-ZnO bandage at different time intervals.

The wound healing potential of the TCS-Alg-ZnO was studied using a murine model and was compared with CS-Alg-ZnO and commercial bandage. A visual recovery of wound healing was recorded photographically for 28 days as shown in Fig 6A–6C Wound closure comparison was done on daily basis. 7 inches wound was created. Until day 7, the wound area was markedly reduced to 3 inches with a TCS-Alg-ZnO bandage as compared to CS-Alg-ZnO bandage in terms of inflammation, remodeling, and skin recovery. At day 28, complete wound closure with no evidence of bandage sticking to skin and exudate was observed with TCS-Alg-ZnO bandage. Results concluded that wound healing capacity and wound closure of the TCS-Alg-ZnO bandage is more enhanced as compared to the other two groups owing to enhanced mucoadhesion and retention of TCS-Alg-ZnO bandage thus ensuring proper dosing, enhanced antimicrobial activity, and high swelling, porosity, and biodegradation due to the presence of increased water content in the form of ZnO-NPs.

### 3.16. Histological evaluation of skin

Histopathological evaluation of skin tissue from the wound area was done to examine the histological changes in skin tissues. Fig 6 shows the initial wound tissues and newly generated skin area after day 7. Assuming the entire length of the wound and epidermal recovery rates at both ends of the wound, the range of epidermal regeneration was calculated [49]. A significant difference in re-epithelialization was observed with TCS-Alg-ZnO bandage. Fig 6D shows wounded tissues treated with Cs-Alg-ZnO bandage and images depict wounded and damaged skin facing dermal and epidermal tearing. Moreover, it showed bleeding wounds and non-vascularization of tissues. Fig 6E indicates wounded tissues healing by a thiolated bandage. It is obvious from images that after stoppage of bleeding, proinflammatory cells migrate towards the wound site and initiates inflammation which is quite visible in TCS-Alg-ZnO bandage treated wounds. In general, macrophages play a very important role in inducing inflammation because of releasing cytokines (inflammation promoters) and additional leucocytes (neutrophils). In the images, it is obvious that inflammatory cells after their repairing from apoptotic cells result in the production of fibroblasts and keratinocytes. In the last two images of Fig 6E proper angiogenesis and tissue regeneration are visible. Hence proved that TCS-Alg-ZnO bandage was far better in healing properties as compared to the unmodified bandage [50].

### 3.17. Biodegradation of the bandage

**Fig 7:**
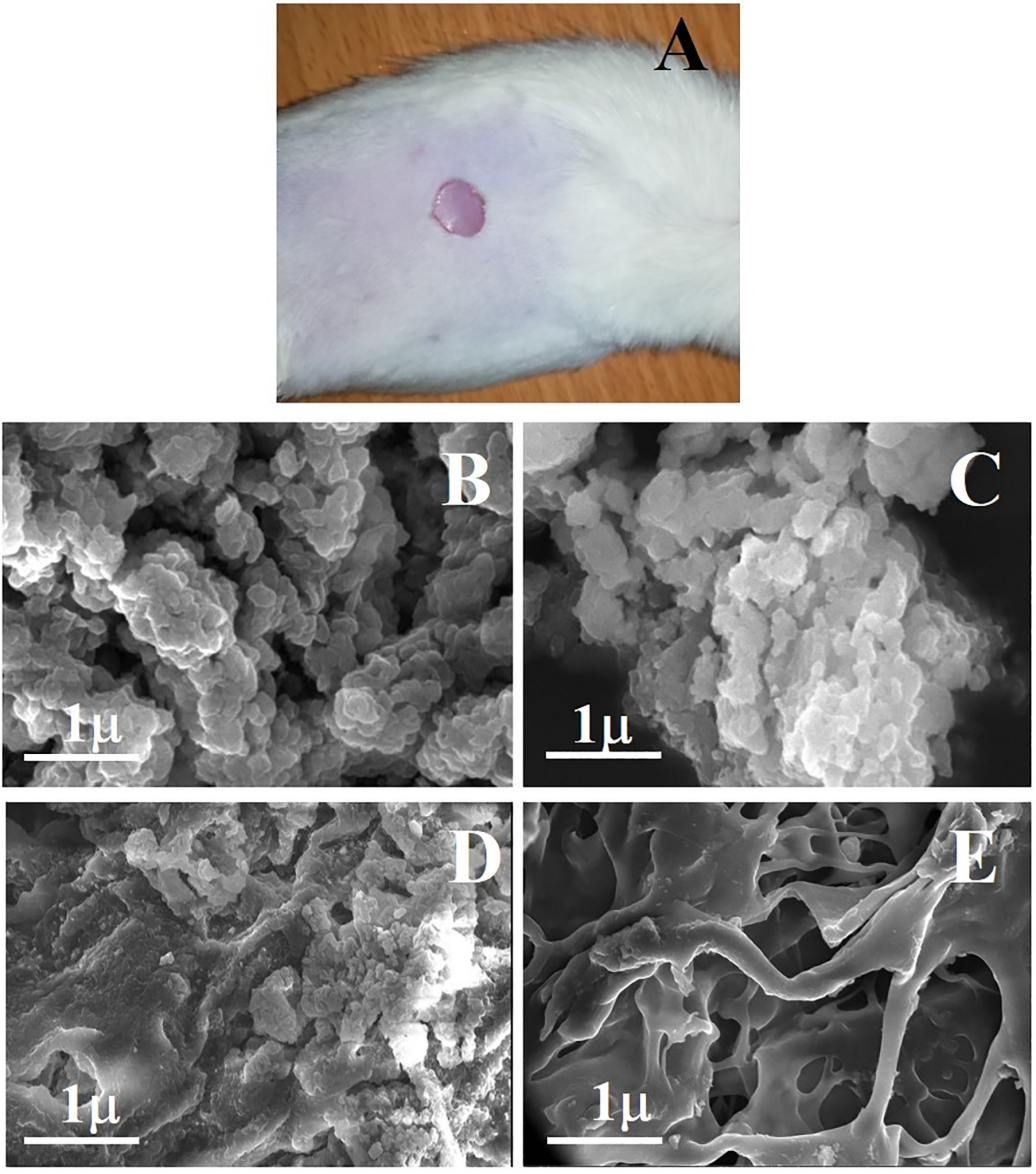
Biodegradation study of thiolated bandage. **(A)** mice with an applied bandage**; (B)** SEM image at 7 days post treatment; **(C)** SEM image at 14 days post treatment; **(D)** SEM image at 21 days post treatment and **(E)** SEM image at 28 days post treatment, showing structural changes in a bandage.

Biodegradation of thiolated bandage was determined visually as well as through SEM analysis. The bandage successfully remained to adhere to the wound till 28 days, but morphological changes were evident during this curse. Bandage appeared as smaller flakes like structures which covered the wound during the progression of the healing process. This facilitated the process of healing as it remained attached to the open part of wound due to strong mucoadhesion. This might provide a better option as wound dressing as ordinary dressing, once applied, sticks to the wound and causes pain when removed forcefully. SEM analysis was performed after every 7 days to see the structural changes in a bandage. As shown in Fig 7A, that after 7 days, bandage started degradation into larger flakes like structures which further reduced in size n 14 days (Fig 7B). after 21 days more than 80% of the bandage was found scattered into small pieces (Fig 7C) and after 28 days it appeared as small particle-like structure (Fig 7D). The results suggested that improved role of the thiolated bandage in protecting and facilitating the wound healing process with self-degradation of the carbohydrate polymers due to its innate increased polymeric strength, increased porosity, and swelling features.

## 4. Conclusion

The present work successfully highlighted the improved prevention and management of post-operative surgical site infections *via* novel bioactive TCS-Alg-ZnO bandage. *In-vitro* characterization of the bandages confirmed higher porosity, improved swelling index, effective biodegradation, enhanced the hemostatic effect of the TCS-Alg-ZnO bandage compared to the commercial wound dressing. *In-vivo* testing resulted in superior wound healing and re-epithelialization of skin at the site of the wound. Hence, TCS-Alg-ZnO with significant higher potential for wound healing can be considered for further investigation and development as a biocompatible and bioactive bandage against surgical site infection.
